# Exploring faculty development initiatives in medical education in resource-limited settings: perspectives and challenges

**DOI:** 10.1186/s12909-025-07848-7

**Published:** 2025-10-27

**Authors:** Arwa I. Ahmed, Alan M. Batt

**Affiliations:** 1 Ontario Centre for Learning, Research and Innovation in Long-Term Care, Baycrest Academy for Research and Education, Toronto, Canada; 2 Dept of Paramedicine, Paramedicine Program Lead, School of Nursing, Institute of Health Policy, Management and Evaluation, Monash University, Queen’s University, University of Toronto, Toronto, Canada

**Keywords:** Faculty development program, Resource-limited setting, Health professionals, Perspectives, Challenges

## Abstract

**Background:**

Faculty Development Programs (FDPs) are integral to institutional priorities to support staff members in leveraging the skills necessary to deliver quality education and enhance the overall learning experience. Little is known about their impact in resource-limited settings. Therefore, the objective of this study was to evaluate the perceptions of medical and health faculty members in Sudan toward FDPs by exploring their views on their performance, the learning environment, and the challenges hindering program implementation.

**Method:**

A descriptive, cross-sectional survey consisting of twenty-six items was used to collect data from faculty members to assess their perceptions of the FDPs.

**Result:**

There was a 77% response rate (*n* = 103) to the survey from the targeted sample size of 134. Most of the staff members (90.3%, *n* = 93) perceive FDP activities as beneficial for enhancing their teaching abilities, while 70.9% (*n* = 73) see improvement in research practices, and 54.4% (*n* = 56) observe benefits to their clinical skills. Fewer respondents (46.6%, *n* = 48) reported improvements in their scientific publications. However, several challenges were identified, with time constraints perceived as a major obstacle to effective program implementation.

**Conclusion:**

In a resource-limited setting, evaluating the program’s effectiveness plays a pivotal role in improving its activities. Providing additional resources, enhancing institutional support, and improving accessibility to activities can strengthen the program’s success, ultimately benefiting both staff and students. These insights may offer valuable guidance for institutions facing similar constraints.

**Supplementary Information:**

The online version contains supplementary material available at 10.1186/s12909-025-07848-7.

## Introduction

Health professions educators are expected to fulfill a variety of roles; they are not merely information providers. They also act as mentors, researchers, managers, administrators, evaluators, and facilitators [1]. While many are typically well-prepared for their clinical responsibilities, few receive formal preparation for their teaching duties [2]. This is despite evidence that in order to excel as educators, faculty members require educational skills in addition to their expertise in their respective disciplines [3]. Faculty development (FD) refers to any planned activities that foster the faculty’s ability to excel in all aspects of their academic professions by improving knowledge and skills to achieve sustainable behavioral change [4–6]. These activities are often provided in the form of seminars, workshops, short courses, on-site visits, fellowships, and other long-term programs [7]. In addition, faculty development programs (FDPs) exhibit considerable diversity across institutions, encompassing a range of both formal and informal contributions [8]. Contemporary FDPs have evolved from focusing on traditional technical and professional competence within disciplines to now encompassing broader aspects such as faculty well-being, institutional quality of life, and personal and professional growth opportunities [9].

The benefits of FDPs are numerous. They improve community-based education, problem-based learning, integration between basic and clinical sciences, student-centered education, comprehensive evaluation, and evidence-based medicine [10]. In addition, FDPs support improved student performance [11], promote humanistic teaching and role modeling [12], and enhance faculty skills in curriculum support, teaching, assessment, organizational leadership, and mentoring [13]. Participating in FD activities results in increased staff satisfaction, confidence, and enhancements in teaching abilities among faculty members [7, 14, 15].

However, several potential obstacles may hinder the implementation of FDPs, including limited financial investment [16], a shortage of training personnel [17], and the lack of comprehensive program design [18]. Barriers to participation in FDPs are often attributed to faculty misconceptions that underestimate the importance of a program or its potential benefits, the belief that clinical skills are more valuable than teaching skills [19], as well as most teachers are unsure about dedicating time to their teaching excellence [20]. Moreover, a lack of accountability, direction, and feedback from the institute’s leadership about teaching performance, and organizational and logistical issues, such as the relevance and applicability of topics to practice, the quality of presenters, advertisement methods (e.g., lack of engaging titles and descriptions), event location and timing were significant barriers to participation in the activities [21].

According to The Accreditation Council for Graduate Medical Education (ACGME), program evaluation is defined as the “*Systematic collection and analysis of information related to the design*,* implementation*,* and outcomes of a graduate medical education program for monitoring and improving the quality and effectiveness of the program* [22]”. The purpose of program evaluation is to judge the value or worth of educational programs [23], with a primary focus on change that has occurred and its impact not only on the learners but also on the teachers, administrators, and stakeholders [24]. In addition, evaluation aims to generate reliable and valid data, which aids curriculum developers in modifying their programs to suit the evolving context and assists researchers in medical education in producing knowledge that can ‘inform the efforts of others’ [25].

Despite the availability of program evaluation models, literature exploring FDP evaluation is scarce. This can be attributed to the innovative nature and unpredictable outcomes of FDPs, which are often difficult to measure using the available models [26]. This concern is underpinned by complexity, which emphasizes the uncertainty and ambiguity of FDPs [27]. Identifying these uncertainties has been a target of researchers by studying the factors that shape the program, such as participants’ characteristics, the influence of stakeholders, evolving knowledge, and patterns in professional practice. In addition, examining the relationship between the program’s elements by considering its context can provide valuable insights into the effectiveness of faculty development initiatives [24]. According to Haji et al. (2013), evaluating FDPs requires considering the program’s context, process, and theory, moving beyond the simplistic question of whether a program worked (outcome-based evaluation) [25].

Recently, others have conceptualized complex interventions in health professions education (HPE) as flexible applications based on principles rather than standard elements [28]. Standard elements function as mechanisms for conveying the underlying principles or theories. In clinical practice, standard elements are defined as details that allow replication of a study with a high level of ‘sameness’, while in complex interventions such as educational practice, they may need to adapt to different contexts [29]. Instead of standardizing activities (e.g., the instructional method of the workshop), the focus should be on steps or processes that these components are intended to facilitate in achieving specific objectives (e.g., engaging participants or enhancing skills) [30]. This principle-focused evaluation, a novel approach introduced by Patton (2017), assesses the value of the program based on adherence to guiding principles rather than the achievement of predetermined goals. Patton’s guiding principle is a statement that offers direction on how to think or act to achieve a specific goal, drawing on personal values, beliefs, and experiences. This strategy offers flexibility in application which enhances creativity in HPE programs. Moreover, it helps prevent rushing to judgments about a program’s worth or success [31, 32].

While faculty development is a recognized and well-researched process globally, it is still evolving within the context of developing countries such as Sudan. This may be attributed to the shortage of expert education scientists, insufficient resources, and/or a lack of institutional priorities [10, 33]. Within Sudan, the setting for the current study, FD remains underdeveloped due to a combination of the above issues [34–36]. Without an understanding of the unique barriers to FDPs in resource-limited settings, we may fail to critically engage with foundational issues in the design, development, implementation, and evaluation of faculty development in such contexts.

In a challenging resource-limited setting, implementing FDPs faces barriers such as resistance to pedagogical changes, overlapping responsibilities in health care, coupled with insufficient administrative support [20]. Additionally, the shortage of both human and financial resources for training is considered a major obstacle [37]. This has resulted in teachers who conform to classical teaching methods without incorporating modern approaches, such as problem-based learning and team-based learning while demonstrating a lack of self-driven learning skills. In addition, there is little research in the field of education, a shortage of community service programs, and insufficient time allocated for professional development within Sudan [38]. We must ensure that FDPs implemented in such resource-limited settings are appropriately developed, researched, and evaluated.

### Conceptual framework

**Fig. 1:**
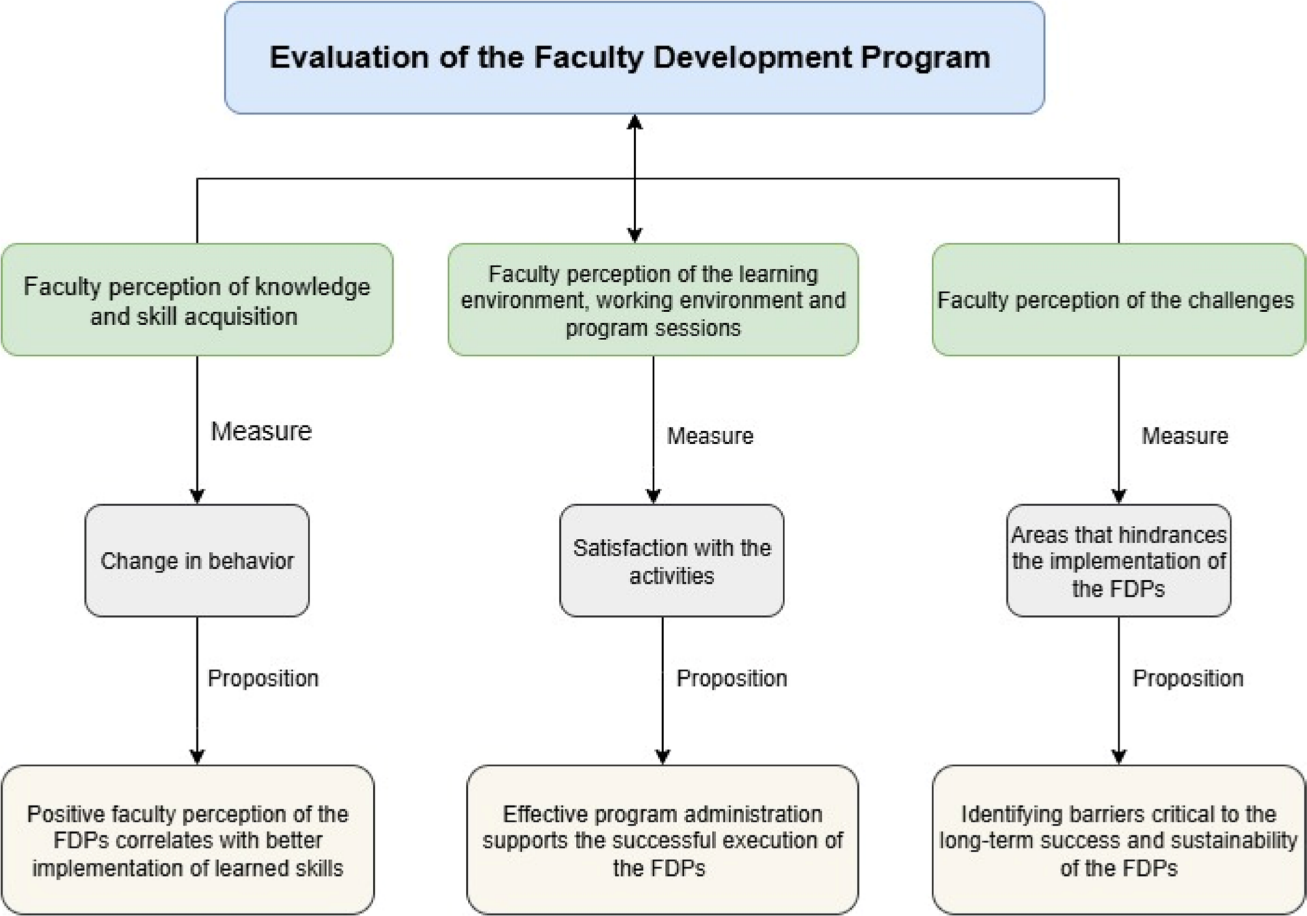
Evaluation Framework of Faculty Perspectives Toward the FDP

Our systemic evaluation adopts the framework by Charlier and Limbert (2019) for evaluating faculty development programs [39], which assesses the effect of working and learning environments on participants’ professional development and teamwork (Fig. 1). The following adaptations were made for the current study in the Sudanese context:

1. We modified the categories of perceived individual learning effects to include the following aspects: teaching skills, clinical skills, student assessment abilities, research practice, scientific publications, collaboration, career development, and commitment to the institution.
2. We added a section to explore participants’ satisfaction with the program sessions, along with the working and learning environment.
3. We introduced a new section to explore the challenges encountered during the implementation of the FDP in resource-limited settings.

The conceptual framework for evaluating faculty perspectives of the FDPs in the present study examines faculty perceptions in three key areas: (1) knowledge and skill acquisition (perceived learning effect) measured by self-reported change in behavior, with positive perceptions reflecting effective application of the learned skills; (2) Perception of the learning environment, working environment, and program sessions measure staff satisfaction with the activities, where effective administration support reflects successful FDP execution. And lastly, (3) challenges are assessed by identifying barriers to FDP implementation, with the goal of addressing these obstacles to improve program outcomes.

This research aims to inform a principles-based framework for assessing faculty perspectives of FDPs in educational institutions in resource-limited settings. It examines participants’ self-perception regarding the impact of program activities on their performance and evaluates the educational environment. In addition, it assesses how participants perceive the applicability of the acquired skills in the learning environment, evaluates their views on session effectiveness, and identifies their perceptions of implementation challenges.

Given these objectives, this study sought to answer the following research questions: How do faculty members in resource-limited settings perceive the impact of Faculty Development Programs (FDPs) on their professional performance and teaching environment? Additionally, what challenges do faculty members face in implementing FDP-acquired skills within their institutions?

The justification for undertaking this study is rooted in the scarcity of information in medical literature, particularly the absence of published studies regarding faculty development in Sudan. This stresses the need to assess the extent to which faculty staff receive developmental training and their perspectives toward faculty development programs. Such insights will not only inform administrators and policymakers about the faculty’s needs but also guide the development of FDPs with an appropriate standard in resource-limited settings.

## Methods

### Study design

This study adopted a descriptive cross-sectional survey design and is reported following the CROSS (Checklist for Reporting of Survey Studies) guidelines [40]. The design allows for a detailed exploration of the status of the FDPs and their impact on staff academic practice.

### Setting

Sudan, one of the largest countries in Africa, is famous for its diverse population and geographic extremes [41]. There has been a transformative change in medical education in Sudan, dating back to 1978 when an Educational Development Centre (EDC) was established at the Faculty of Medicine, University of Gezira, which is considered a pioneer in the introduction of community-based education in Sudan [42]. In 1980, the EDC at the Faculty of Medicine, University of Khartoum significantly offered teacher training programs about innovative instructional and evaluation methods, providing consultation on curricula development for medical institutes, and fostering a culture of research and scientific publication. In addition, EDC trained paramedical teaching staff and supported primary health care programs in Sudan [43]. There has been a growth in the number of centers of continuous professional development in both the public and private sectors across Sudan in recent years. Overall, the nation’s medical education system has shown resilience over time, continually adapting to meet both local demands and universal health problems, even in light of the ongoing conflict.

The Center for Professional Development (CPD) was established at the National University in December 2009 due to the college’s rapid growth and expansion. The center’s vision is to be a leading hub for training and development programs, and its mission is to upgrade staff abilities and enhance educational service quality. Objectives include promoting continuous education, introducing innovative teaching concepts, supporting research practice, fostering the knowledge and skills of staff to pursue their academic career, and establishing an interactive online support platform to facilitate online education.

Activities are determined based on staff needs, including workshops, journal clubs, advice sessions, and lectures. Its policy focuses on providing comprehensive training to ensure staff members are equipped with updated skills aligned with the university’s vision and mission while adhering to high-quality standards.

### Participants

The target population comprised permanent staff members in the medical and health faculties of the National University, with no exclusion criteria.

A Stratified Sampling Technique was employed to ensure representation across all faculties. Each faculty was treated as a stratum, and random sampling for faculty members within each stratum (faculty) was performed using the research randomizer (https://www.randomizer.org/).

The required sample size was 134, calculated using the Yamen sample-size equation, applying a 95% confidence interval and a 0.05 margin of error. Proportional allocation was used to determine the number of participants in each faculty, based on the total number of staff members in that faculty.

### Data collection method

Data were collected using a secure Google Form between September 2022 -March 2023. The study questionnaire consisted of twenty-six questions across two sections: the first section recorded demographic data (six questions), and the second section (twenty questions) explored the details of the faculty development programs, covering staff perceptions in the following five domains:

1. Perceived learning effect.
2. Perception of the learning environment.
3. Perception of the working environment.
4. Perception of the program activities.
5. Challenges facing the implementation of the FDPs.

The first three domains were directly informed by the systemic framework for the evaluation of a faculty development program by Charlier and Lambert (2019) [39], while the remaining two domains were designed by the research team to better answer the research questions. Participants responded to the questions in section two using a 5-point Likert scale from ‘strongly disagree’ [1] to ‘strongly agree’ [5] (Annex I).

### Data analysis

Categorical data are presented as frequencies and percentages. Numerical data are presented as mean and standard deviation values. The student’s t-test was used to compare between two groups. A one-way ANOVA test was used to compare more than two groups. Bonferroni’s post-hoc test was used for pair-wise comparisons when the ANOVA test was significant. The significance level was set at *P* ≤ 0.05. Statistical analysis was performed with IBM SPSS Statistics for Windows, Version 23.0. Armonk, NY: IBM Corp.

### Study preparation

The researchers conducted a review of documents related to implemented programs and Continuing Professional Development (CPD) strategic plans. To ensure content validity, the questionnaire was evaluated by key stakeholders, including the university’s Vice President, the Dean of Graduate Studies, and the CPD Director. Based on their input, necessary refinements were made to enhance the instrument’s validity. The internal consistency of the questionnaire was assessed using Cronbach’s alpha, which yielded a value of 0.829. A list of the staff members, along with their institutional contact details (email and/or phone numbers), was obtained from the university administration. The researcher then distributed the survey, providing a clear explanation of the study’s purpose and assuring participants that their responses would be kept confidential.

## Results

### Demographic data

The present study enrolled 103 participants: 31 males (30.1%) and 72 females (69.9%). The most prevalent age category was 30–39 years old (37.9%), followed by 40–49 years old (31.1%), while the least common age category was less than 30 years old (12.6%). Approximately half of the participants (47.6%) had a Master’s (MSc) degree, about one-quarter of participants (23.3%) had a Medical Doctorate (MD), the same percentage was found in participants with a PhD, and the lowest percentage (4.9%) was participants with a Fellowship. Only one participant reported other qualifications. The most prevalent field of profession was Medicine (37.9%), followed by Pharmacy (15.5%), while the least prevalent field was Nursing (1.9%). Approximately half of the participants (47.3%) were lecturers, one-fifth (19.8%) were assistant professors, 13.6% were associate professors, and only 4.9% were professors. Approximately one-third of participants (34%) had more than 10 years of experience, 21.4% had 8–10 years of experience, and the lowest percentage (11.7%) was reported for participants with 3–5 years of experience (Table 1).

**Table 1:** Frequencies (n) and percentages (%) for demographic data of the study participants (*N* = 103)

Demographic data	*n*	%
Gender		
Male	31	30.1
Female	72	69.9
Age		
less than 30 years	13	12.6
30–39 Years	39	37.9
40–49 Years	32	31.1
50 years and above	19	18.4
Qualification		
MSc	49	47.6
MD	24	23.3
PhD	24	23.3
Fellowship	5	4.9
Other	1	1
Field of profession		
Medicine	39	37.9
Dentistry	14	13.6
Pharmacy	16	15.5
Medical laboratory	15	14.6
Nursing	2	1.9
Radiology	12	11.7
Physiotherapy	5	4.9
Academic rank		
Lecturer	50	48.5
Assistant professor	34	33
Associate professor	14	13.6
Professor	5	4.9
Experience		
1–3 Years	14	13.6
3–5 Years	12	11.7
5–7 years	20	19.4
8–10 Years	22	21.4
More than 10 years	35	34

### Perception of the faculty development programs

Frequencies and percentages of responses to questions regarding the FDPs are presented in Table 2.

**Table 2:** Frequencies (*n*) and percentages (%) for responses to the fdp’s questions (*N* = 103)

Item		Strongly agree	Agree	Neutral	Disagree	Strongly disagree
Q1.My teaching skills improved after the completion of the faculty development programs	*n*	34	59	7	2	1
	%	33%	57.3%	6.8%	1.9%	1%
Q2.My clinical skills improved after the completion of the faculty development programs	*n*	16	40	38	6	3
	%	15.5%	38.8%	36.9%	5.8%	2.9%
Q3. My student assessment abilities improved after the completion of the faculty development programs	*n*	29	56	14	3	1
	%	28.2%	54.4%	13.6%	2.9%	1%
Q4.My research practice abilities improved after the completion of the faculty development programs	*n*	26	47	25	3	2
	%	25.2%	45.6%	24.3%	2.9%	1.9%
Q5.My Scientific publications improved after the completion of the faculty development programs	*n*	14	34	45	8	2
	%	13.6%	33%	43.7%	7.8%	1.9%
Q6.The programs enhance my skills in collaborative work	*n*	21	65	15	1	1
	%	20.4%	63.1%	14.6%	1%	1%
Q7.Faculty development programs positively improve my career	*n*	30	58	12	2	1
	%	29.1%	56.3%	11.7%	1.9%	1%
Q8. Faculty development programs increase my commitment to my institute	*n*	27	50	22	2	2
	%	26.2%	48.5%	21.4%	1.9%	1.9%

The mean and standard deviation (SD) values for the response scores were 3.9 (0.68).

### Perception of the learning environment

Frequencies and percentages of responses to questions regarding the perception of the learning environment are presented in Table 3.

**Table 3:** Frequencies (*n*) and percentages (%) for responses to perception of the learning environment questions (*N* = 103)

Item		Strongly agree	Agree	Neutral	Disagree	Strongly disagree
Q1 Organizational tools are provided (e.g., guidelines, calendar, and objectives) before conducting the programs	*n*	34	48	12	6	3
	%	33%	46.6%	11.7%	5.8%	2.9%
Q2 Programs are carried out according to the schedules provided	n	23	60	13	5	2
	**%**	22.3%	58.3%	12.6%	4.9%	1.9%

The mean and standard deviation (SD) values for the response scores were 3.9 (1.03).

### Perception of the working environment

Frequencies and percentages of responses to the question regarding the perception of the working environment are presented in Table 4.

**Table 4:** Frequencies (*n*) and percentages (%) for responses to the perception of the working environment questions (*N* = 103)

Item		Strongly agree	Agree	Neutral	Disagree	Strongly disagree
Q1 The working environment allowed me to apply the skills I gained after completing the faculty development programs	*n*	34	48	12	6	3
	%	33%	46.6%	11.7%	5.8%	2.9%

The mean and standard deviation (SD) scores for the response scores were 3.6 (1.17).

### Perception of the program sessions

Frequencies and percentages of responses to questions regarding perception of the program sessions are presented in Table 5.

**Table 5:** Frequencies (*n*) and percentages (%) for responses to the perception of the program sessions questions (*N* = 103)

Item		Strongly agree	Agree	Neutral	Disagree	Strongly disagree
Q1 It is convenient for me to participate in the sessions	*n*	14	65	21	3	0
	%	13.6%	63.1%	20.4%	2.9%	0%
Q2 I found myself engaged during the sessions	*n*	18	61	20	3	1
	%	17.5%	59.2%	19.4%	2.9%	1%
Q3 I am satisfied with the speakers’ performance	*n*	17	66	18	2	0
	%	16.5%	64.1%	17.5%	1.9%	0%
Q4 The sessions were effective in encouraging me to evaluate my understanding (e.g., feedback, post-test, response to questions asked) of the topic and to fill any gaps identified	*n*	23	61	17	2	0
	%	22.3%	59.2%	16.5%	1.9%	0%
Q5 The sessions suit my educational needs	*n*	12	71	16	4	0
	%	11.7%	68.9%	15.5%	3.9%	0%

The mean and standard deviation (SD) values for the response scores were 3.9 (0.59).

### Challenges facing the implementation of the faculty development programs

Frequencies and percentages of responses to questions regarding the challenges facing the implementation of the FDPs are presented in Table 6.

**Table 6:** Frequencies (*n*) and percentages (%) for responses to challenges facing the implementation of the FDPs questions (*N* = 103)

Challenge		Strongly agree	Agree	Neutral	Disagree	Strongly disagree
Q1.Lack of administrative support	*n*	3	31	36	28	5
	%	2.9%	30.1%	35%	27.2%	4.9%
Q2.Time limits and busy schedules	*n*	25	44	23	11	0
	%	24.3%	42.7%	22.3%	10.7%	0%
Q3.Financial constraints	*n*	12	38	26	21	6
	%	11.7%	36.9%	25.2%	20.4%	5.8%
Q4.Lack of awareness	*n*	8	10	29	44	12
	%	7.8%	9.7%	28.2%	42.7%	11.7%

Approximately two-thirds of participants (67%) strongly agreed or agreed that the most important challenge is time limits and busy schedules. This was followed by financial constraints (48.6%), a lack of administrative support (33%), and finally a lack of awareness about the FDPs (17.5%).

### Association between gender and perceptions of the faculty development programs

**Fig. 2:**
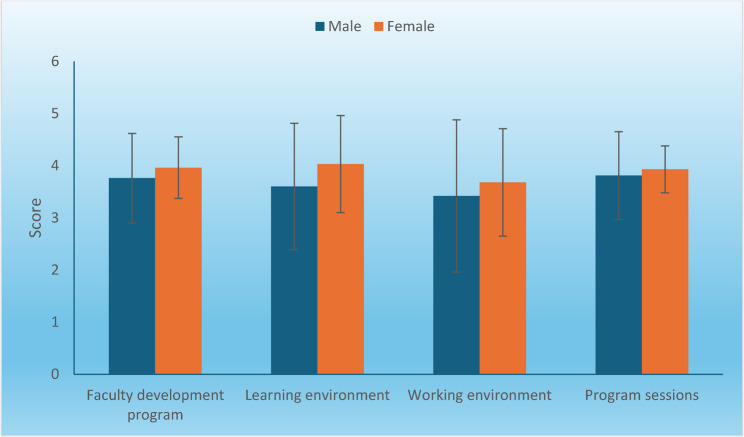
Bar chart representing the mean and standard deviation values of perception scores for males and females

There was no statistically significant difference between the perceptions of the FDPs, the learning environment, the working environment, and the program sessions in males and females (Table 7; Fig. 2).

**Table 7:** Mean, standard deviation (SD) values, and results of student’s t-test for comparison between the perception of the FDPs in males and females

Perception items	Male (*n* = 31)		Female (*n* = 72)		*P*-value
	Mean	SD	Mean	SD	
Faculty development program	3.76	0.86	3.96	0.59	0.243
Learning environment	3.60	1.21	4.03	0.93	0.051
Working environment	3.42	1.46	3.68	1.03	0.370
Program sessions	3.81	0.84	3.93	0.45	0.466

* Significant at *P* ≤ 0.05

### Association between age category and perceptions of the faculty development programs

**Fig. 3:**
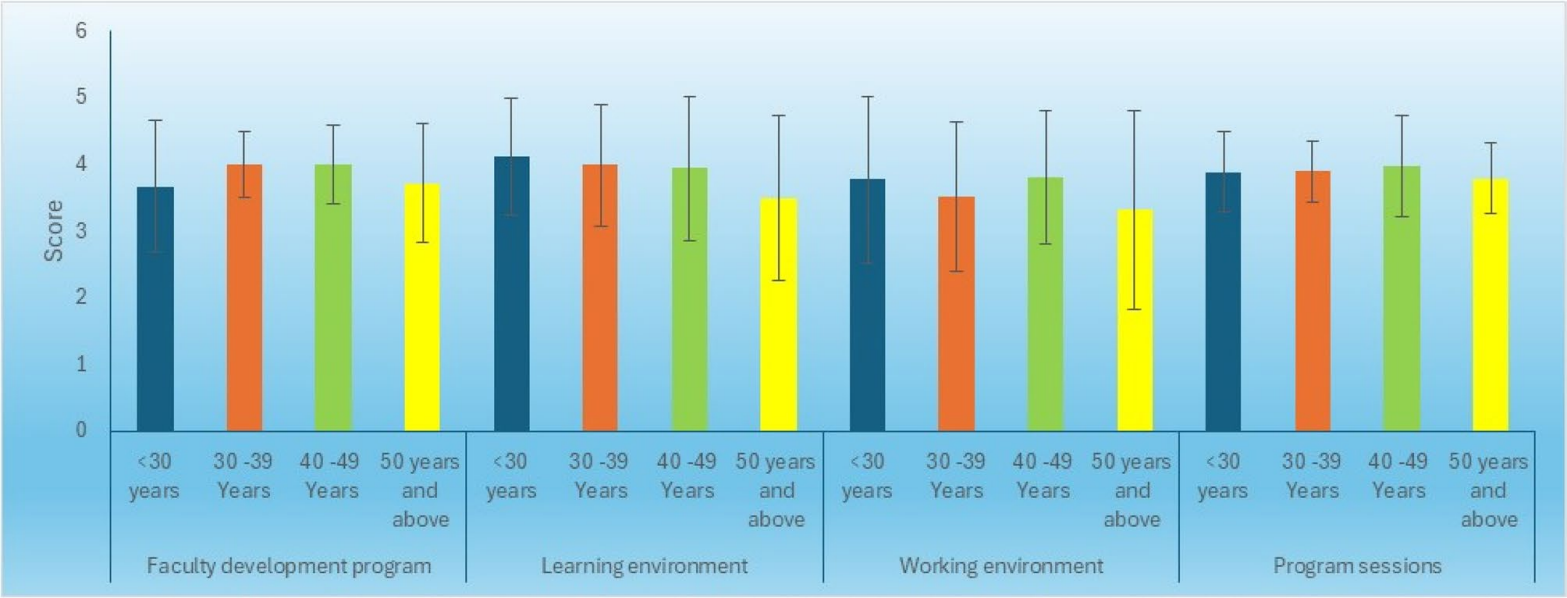
Bar chart representing the mean and standard deviation values of perception scores among participants across different age categories

There was no statistically significant difference between the perception of the FDPs, the learning environment, the working environment, and the perception of program sessions in participants with different age categories (Table 8; Fig. 3).

**Table 8:** Mean, standard deviation (SD) values, and results of One-Way ANOVA test for comparison between the perception of the FDPs in different age categories

Perception items	Age category	Mean		SD		*P*-value	
Faculty development program	Less than 30 years (*n* = 13)	3.67	0.98		0.276		
	30–39 Years (*n* = 39)	3.99	0.49				
	40–49 Years (*n* = 32)	3.99	0.59				
	50 years and above (*n* = 19)	3.72	0.90				
Learning environment	Less than 30 years (*n* = 13)	4.12	0.87		0.294		
	30–39 Years (*n* = 39)	3.99	0.92				
	40–49 Years (*n* = 32)	3.94	1.08				
	50 years and above (*n* = 19)	3.50	1.24				
Working environment	Less than 30 years (*n* = 13)	3.77	1.24		0.455		
	30–39 Years (*n* = 39)	3.51	1.12				
	40–49 Years (*n* = 32)	3.81	1.00				
	50 years and above (*n* = 19)	3.32	1.49				
Program sessions	Less than 30 years (*n* = 13)	3.88	0.60		0.755		
	30–39 Years (*n* = 39)	3.89	0.45				
	40–49 Years (*n* = 32)	3.98	0.76				
	50 years and above (*n* = 19)	3.79	0.53				

*Significant at *P* ≤ 0.05

### Association between qualifications and perceptions of the faculty development program

There was no statistically significant difference in the perception of the FDPs, the learning environment, the working environment, and the perception of program sessions.

## Discussion

We sought to understand the perception of FDPs among staff in health and medical faculties. Specifically, we investigated their perspectives on knowledge acquisition, their views of the learning and working environment, and the challenges they believe affected the program’s implementation. We reviewed previous research [44–47] aimed at exploring staff perceptions of FDP in a healthcare setting and found that participants generally perceive FDP as valuable for improving their professional roles which aligns with our study.

Applying the conceptual framework, we observed that participants’ high perception of knowledge and skill acquisition serves as a measure for behavior change. We propose that positive perceptions indicate that participants are more likely to apply the skills they’ve learned in their work. This claim is supported by Lacruz et al. (2019), who examined the impact of a professional training program on workplace competence. Their study demonstrated a significant relationship between satisfaction with the applicability of knowledge and skills learned and improvements in professional competence, with the vast majority of participants being satisfied with the course and believing they could apply the new knowledge and skills at work [48]. Similarly, Singh et al. (2014) emphasize that the goal of faculty development is to facilitate the application of skills in the workplace, as there is a growing focus on the “transfer of training” among health professionals. Our study indicates that participants’ attitudes toward the training significantly impact their willingness to engage with the program and apply what they have learned. Attitude toward behavior is defined as the positive or negative feelings an individual has regarding a specific behavior. This attitude is shaped by their beliefs about performing the behavior and their evaluation of the associated outcomes [49]. Thus, a positive attitude (perception) of participants towards FD suggests they are more likely to transfer the knowledge and skills gained during the program into their practice, ultimately contributing to the program’s success and its impact on student learning outcomes.

Our study findings highlighted that a significant number of participants reported improved teaching performance, which aligns with previous research showing that health professionals enhance their teaching skills [50] and student assessment abilities after completing the FDP activities [51]. While clinical and research practice abilities were moderately perceived among the participants, scientific publication perception was lower, with nearly half of the participants responding neutrally. These findings were supported by Lee et al. (2010), who attributed low research output to the lack of structured research training, insufficient mentorship, and inadequate institutional support [52]. This variation in perceived impact may also reflect the design focus of the FDPs, which traditionally prioritize teaching competencies. Research-related activities often require more time and sustained support, which are major obstacles in resource-limited settings [53]. On the other hand, collaborative skills and overall career improvement were positively perceived; this underscores the value of faculty development in fostering collaboration as a core skill in achieving long-term change in health education. By enhancing these collaborative skills, FDPs not only contribute to individual career growth but also facilitate the successful implementation of educational innovations in clinical settings. Furthermore, the findings illustrate that FDPs not only enhanced knowledge and skill acquisition but also increased participants’ commitment to their institute, consistent with Campion et al. (2016), who illustrated that participants felt more committed to their institution after engaging in FDP activities, perceiving this commitment because of the institution’s investment in their professional growth [54]. The FDP fostered stronger institutional connectivity amongst faculty members, suggesting that effective development programs may contribute to both professional growth and improved institutional loyalty.

Our findings highlighted that most participants reported high satisfaction with the program sessions, program organization, and found the work environment supportive of applying the skills they had gained. This finding corresponds with Burgess et al. (2019) study, who argue that organizational support facilitated alignment between participants’ experiences and the program’s intended outcomes, which in turn enabled active engagement and practical application of the learned skills [6]. This suggests the claim that the satisfaction of the participants observed in the study is closely related to the effective execution of the program.

Identifying the challenges faced by the participants was a crucial part of this study, particularly because it was conducted in a resource-limited setting. By highlighting these barriers, the study stresses the importance of developing strategies that address resource shortages while maintaining program sustainability. Interestingly, time constraints emerged as the most cited challenge; similarly, Puri et al. (2012) identified time as a major factor hindering staff participation in FDPs [55], attributing this challenge to clinical load and administrative tasks [21, 56]. This highlights the need for a flexible schedule for the FDP and the incorporation of asynchronous activities to facilitate greater staff engagement.

While this study offers important insights into faculty perceptions toward FDPs in Sudan, there are several limitations to consider. First, the literature discussing FDPs in resource-limited settings is sparse, and as such, we lack critical insight into the issues that may be faced across different settings. Second, this study does not represent a comprehensive program evaluation, as it primarily focuses on capturing staff perceptions, and did not seek to explore them in detail. We acknowledge the limitations of self-reported measures but offer that, combined with the perceived challenges in this resource-limited context, these provide important insight to inform future activities. The survey design could serve as a part of a larger evaluation program, combined with other methodologies for a more thorough assessment planned in the future. Third, our sample size comprised 134 potential participants, with 103 respondents, which may limit the generalizability of the findings. Finally, the study was conducted at a single institution due to the project’s individual effort, which may fail to capture the diverse experiences of faculty members across different settings. Future research should consider these limitations and aim for a broader, multi-institutional approach to better understand FD needs and experiences in Sudan.

## Conclusion

Understanding staff perceptions of FDPs is critical to the program evaluation process. This study is considered a micro-evaluation framework that aims to provide broad insights into the effectiveness of FDPs, not only at the staff level but also at the institutional level while addressing challenges that hinder standardized implementation. Although the implementation of program evaluation faces challenges in resource-limited settings, this framework can serve as a tool for FDP sponsors that offer a holistic picture of FDP value. Given the participants’ self-perceived positive impact on their behaviors and skills, institutions should continue to invest in FDPs and seek to use diverse and holistic approaches when evaluating FDPs to support objective measurements of improvements in educational quality and professional competencies.

### Recommendations

We recommend that future studies adopt appropriate mixed methods approaches to better understand stakeholders’ priorities and needs for further improvement of the program. We further suggest providing more detailed insights into the challenges encountered when designing surveys, with a clearer distinction between the factors that facilitate faculty participation and those that hinder the implementation of the programs.

## Supplementary Information

Supplementary Material 1.

## Data Availability

All data generated or analyzed during this study are included in this published article.

## Abbreviations

FDPs: Faculty Development Programs
ACGME: Accreditation Council for Graduate Medical Education
HPE: Health Professions Education
CROSS: Checklist for Reporting of Survey Studies
CPD: Center for Professional Development
EDC: Educational Development Centre
MSc: Master of Science
MD: Medical Doctorate
SD: Standard Deviation
n: Frequencies
