# Supplementary material for: Exploring faculty development initiatives in medical education in resource-limited settings: perspectives and challenges
Source: BMC Med Educ. 2025 Oct 27;25:1501. doi: 10.1186/s12909-025-07848-7 (PMC12560284; doi:10.1186/s12909-025-07848-7)
Supplement: Supplementary file 1 — Supplementary Material 1. [file 12909_2025_7848_MOESM1_ESM.docx]

**Annex I:**

**Section 1: Demographic and Professional Details**

- Age:
- Gender: ( 1) Male ( 2) Female
- Qualification:(1) BDS/MBBS (2)MSC ( 3) PhD

(4)MD ( 5) Fellowship

- Field of profession: (1) Medicine (2) Dentistry (3) Pharmacy (4)Radiology (5)Physiotherapy (6) Medical Laboratory (7) Nursing
- Academic Rank: (1)Lecturer (2)Assistant professor

(4)Associate professor (5) Professor

- Teaching Experience (years):
- **Section 2: Perception of the Faculty Development Program**

**Tick the appropriate option**

| **Evaluation category** | **Possible options** | | | | |
| --- | --- | --- | --- | --- | --- |
| **Perception of the Faculty Development Program** | **Strongly disagree** | **Disagree** | **Neutral** | **Agree** | **Strongly agree** |
| 1. My teaching skills improved after the completion of the faculty development programs |  |  |  |  |  |
| 1. My clinical skills improved after the completion of the faculty development programs |  |  |  |  |  |
| 1. My Student assessment abilities improved after the completion of the faculty development programs |  |  |  |  |  |
| 1. My Research practice abilities improved after the completion of the faculty development programs |  |  |  |  |  |
| 1. My Scientific publications improved after the completion of the faculty development programs |  |  |  |  |  |
| 1. The programs enhance my skills in collaborative work |  |  |  |  |  |
| 1. Faculty development programs positively improve my career |  |  |  |  |  |
| 1. Faculty development programs increase my commitment to my institute |  |  |  |  |  |

| **Evaluation category** | **Possible options** | | | | | | |
| --- | --- | --- | --- | --- | --- | --- | --- |
| **Perception of the learning environment** | **Strongly disagree** | **Disagree** | **Neutral** | | **Agree** | | **Strongly agree** |
| 1. Organizational tools are provided (e.g., guidelines, calendar, and objectives) before conducting the programs |  |  |  | |  | |  |
| 1. Programs are carried out according to schedules provided |  |  |  | |  | |  |
| **Evaluation category** | **Possible options** | | | | | | |
| **Perception of the working environment** | **Strongly disagree** | **Disagree** | **Neutral** | **Agree** | | **Strongly agree** | |
| 1. The working environment allowed me to apply the skills I gained after completing the faculty development programs |  |  |  |  | |  | |
| **Evaluation category** | **Possible options** | | | | | | |
| **Perception of the program session** | **Strongly agree** | **Disagree** | **Neutral** | **Agree** | | **Strongly agree** | |
| 1. It is convenient for me to participate in the sessions |  |  |  |  | |  | |
| 1. I found myself engaged during the sessions |  |  |  |  | |  | |
| 1. I am satisfied with the speakers' performance |  |  |  |  | |  | |
| 1. The sessions were effective in encouraging me to evaluate my understanding (eg, feedback, posttest, response to questions asked) of the topic and to fill any gaps identified |  |  |  |  | |  | |
| 1. The sessions suit my educational needs |  |  |  |  | |  | |
| **Evaluation category** | **Possible options** | | | | | | |
| **Challenges facing the implementation of FD programs** | **Strongly disagree** | **Disagree** | **Neutral** | **Agree** | | **Strongly agree** | |
| 1. Lack of administrative support for faculty development programs |  |  |  |  | |  | |
| 1. Time limits and busy teaching schedules affect my ability to receive faculty development programs |  |  |  |  | |  | |
| 1. Financial constraints affect faculty development programs in my institute |  |  |  |  | |  | |
| 1. Lack of awareness about the faculty development programs |  |  |  |  | |  | |
